# Wetland restoration yields dynamic nitrate responses across the Upper Mississippi river basin

**DOI:** 10.1088/2515-7620/ac2125

**Published:** 2021

**Authors:** Grey R Evenson, Heather E Golden, Jay R Christensen, Charles R Lane, Adnan Rajib, Ellen D’Amico, David Tyler Mahoney, Elaheh White, Qiusheng Wu

**Affiliations:** 1 U.S. Environmental Protection Agency, Office of Research and Development, Center for Environmental Measurement and Modeling, Cincinnati, OH, United States of America; 2 U.S. Environmental Protection Agency, Office of Research and Development, Center for Environmental Measurement and Modeling, Athens, GA, United States of America; 3 Department of Environmental Engineering, Frank H. Dotterweich College of Engineering, Texas A&M University, Kingsville, TX, United States of America; 4 Pegasus Corporation c/o U.S. Environmental Protection Agency, Office of Research and Development, Cincinnati, OH, United States of America; 5 Civil and Environmental Engineering Department, University of Louisville, Louisville, KY, United States of America; 6 Oak Ridge Institute for Science and Education c/o U.S. Environmental Protection Agency, Office of Research and Development, Cincinnati, OH, United States of America; 7 Department of Geography, University of Tennessee, Knoxville, TN, United States of America

**Keywords:** nitrogen, denitrification, prioritization, targeting, non-floodplain wetlands, geographically isolated wetlands

## Abstract

Wetland restoration is a primary management option for removing surplus nitrogen draining from agricultural landscapes. However, wetland capacity to mitigate nitrogen losses at large river-basin scales remains uncertain. This is largely due to a limited number of studies that address the cumulative and dynamic effects of restored wetlands across the landscape on downstream nutrient conditions. We analyzed wetland restoration impacts on modeled nitrate dynamics across 279 subbasins comprising the ∼0.5 million km^2^ Upper Mississippi River Basin (UMRB), USA, which covers eight states and houses ∼30 million people. Restoring ∼8,000 km^2^ of wetlands will reduce mean annual nitrate loads to the UMRB outlet by 12%, a substantial improvement over existing conditions but markedly less than widely cited estimates. Our lower wetland efficacy estimates are partly attributed to improved representation of processes not considered by preceding empirical studies – namely the potential for nitrate to bypass wetlands (i.e., via subsurface tile drainage) and be stored or transformed within the river network itself. Our novel findings reveal that wetlands mitigate surplus nitrogen basin-wide, yet they may not be as universally effective in tiled landscapes and because of river network processing.

## 1. Introduction

Excess nutrient loads to landscapes from agricultural intensification and urbanization continue to cause chronic global water quality issues. Surplus nitrogen (i.e., the difference between nitrogen fertilizer inputs and nitrogen removed with harvested crops) in agricultural landscapes is a particular concern because nitrogen’s mobile forms (e.g., nitrate) are readily transported to waterways, contributing to eutrophication, harmful algal blooms, and human health impacts across freshwater and marine systems (Rabalais *et al* 2002, Diaz and Rosenberg 2008). The ∼ 0.5 million km^2^ Upper Mississippi River Basin, USA, is a prominent exemplar of how surplus nitrogen continues to elicit annual hypoxic ‘dead zones’ at the basin’s outlet to the Gulf of Mexico (Turner *et al* 2008). The largely agricultural basin’s degraded aquatic systems also reflect the consequences of large-scale and long-term changes to land use and management (Turner and Rabalais 2003, Van Meter and Basu 2017).

While ‘natural’ wetlands continue to be lost at high rates across the globe (Creed *et al* 2017), wetland restoration and construction are primary management options for mediating degraded water quality. Decades of studies demonstrate wetlands’ strong capacity to remove nitrogen (N) from stormwater and agricultural runoff (Johnston 1991, Kadlec *et al* 2000, Kalcic *et al* 2018) via plant uptake, denitrification, and settling of organic particulates (Seitzinger *et al* 2006, Van Cleemput *et al* 2007). Therefore, programs such as the US Department of Agriculture’s (USDA) Wetland Reserve Easements (Morefield *et al* 2016) and the European Land Conservation Network’s (ELCN) promotion of private land easements (Račinska and Vahtrus 2018) are ubiquitous.

However, uncertainty prevails regarding the potential of wetlands to mitigate nutrient conditions across large river basin scales (Fisher and Acreman 2004, Thorslund *et al* 2017, Hansen *et al* 2018, Golden *et al* 2019). Restored wetlands show considerable potential for cumulatively removing N across landscapes, as demonstrated by studies applying averaged empirical N removal rates from individual wetlands to landscape scales (Mitsch *et al* 2001, Cheng and Basu 2017). This is particularly true if restoration is targeted in high N source areas (Cheng *et al* 2020). These studies provide a solid foundation for understanding the capacity of wetland nitrate removal across landscapes. Yet previous work assumes steady-state or stationary relationships between wetland N removal and in-stream N concentrations – relationships that typically do not exist in watersheds, i.e., dynamic, nonstationary systems.

A clear gap therefore remains in connecting dynamic landscape wetland N removal to in-stream responses, particularly across large river basins. We pose a critical scientific question to fill this niche: What are the quantifiable links between cumulative landscape-scale wetland N removal and changes in N-based water quality across large river basins (Figure S1 (available online at stacks.iop.org/ERC/3/095002/mmedia)) – both at the river basin outlet and at the scale of its contributing subbasins? This question remains unanswered, in part, because – to our knowledge – no process-based model exists that explicitly integrates landscape wetland N dynamics to assess the in-stream N response to wetland restoration across large spatial extents.

Our goal was therefore to simulate the potential of wetland restoration to reduce nitrate as N (${\text{NO}}_{3}^{-}-\text{N}$, hereafter ${\text{NO}}_{3}^{-}$) yields, loads, and concentrations across 279 subbasins and the outlet of the ∼0.5 million km^2^ Upper Mississippi River Basin (UMRB), an agriculturally intensive basin that is the largest contributor of excess nitrogen to the Gulf of Mexico hypoxic ‘dead zones’ (Crawford *et al* 2019). The basin’s poor water quality conditions have been attributed to the use of synthetic fertilizers for agricultural production as well as the historical loss of wetlands to agricultural development and urbanization. Additionally, a substantial and increasing portion of the basin is artificially drained via tiles (i.e., subsurface drainage pipes), which remove water from soils for increased agricultural production while facilitating the rapid transport of nitrate off the land and into downstream waters (David and Gentry 2000, Tomer *et al* 2003, David *et al* 2010).

We specifically assessed how the location and relative magnitude of subbasin (or ‘local’) scale wetland restoration across the UMRB affected ${\text{NO}}_{3}^{-}$ yields, loads, and concentrations at the river basin outlet. We did this by: (1) building and calibrating a river basin scale process-based model of the UMRB that explicitly accounts for wetland hydrologic and nutrient cycling dynamics – and those throughout the river network, (2) simulating the restoration of the wetlands outside of floodplains (i.e. non-floodplain wetlands), and (3) evaluating changes in ${\text{NO}}_{3}^{-}$ yields, loads, and concentrations between our baseline and restoration models – at both subbasin and river basin outlets. Our approach provides new insights into how nitrate responds to wetland restoration across large river basins because it explicitly simulates wetland water and nutrient cycling – unlike other stationary empirical approaches or large river basin models (Golden *et al* 2019).

## 2. Methods

### 2.1. Baseline model development

We used the Soil and Water Assessment Tool (SWAT) model (version 659) to assess the effects of wetland restoration on changes in ${\text{NO}}_{3}^{-}$ yields, loads, and concentrations across the UMRB (Figure S2). SWAT is a process-based watershed-scale hydrologic model commonly applied to simulate management impacts on hydrologic flows and aquatic nutrients (Gassman *et al* 2007). The model discretizes a watershed into a series of subbasins, which are composed of one or more hydrologic response units (HRUs), or areas of similar soil, slope, and land use properties, and simulates a daily water and nutrient balance for each HRU (Neitsch *et al* 2011). Transformation of nitrogen in the soil is controlled by partial differential equations that represent the nitrogen cycle (e.g., N_2_ fixation, N plant uptake, denitrification, leaching, and volatilization; see Text S1 for further details).

Each HRU’s simulated daily streamflow and nutrient loads then enter the river network, where nutrients are stored and processed (i.e., removed permanently or cycled through the river system) and are routed progressively downstream to the basin outlet (Neitsch *et al* 2011). In-stream transformation of nitrogen in the model is governed by partial differential equations representing growth and decay of algae, biological oxidation rates for different nitrogen species, water temperature, and settling of organic N with sediment (see Text S1).

**Figure 1.**
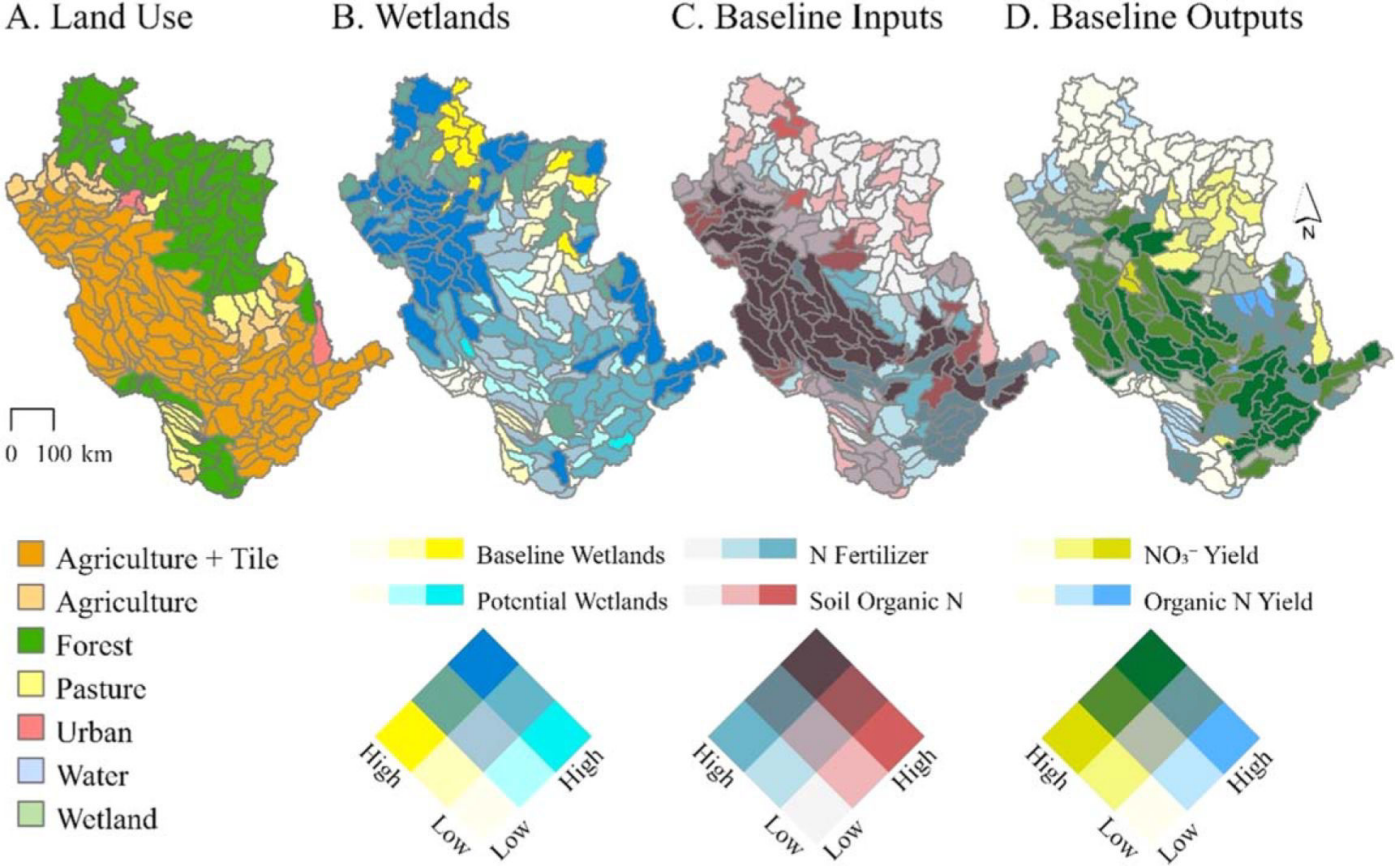
Spatial distribution and magnitudes of the primary inputs and outputs for the Upper Mississippi River Basin (UMRB) SWAT model. For each of the model’s subbasins (n=279), the figure shows (A) the dominant land use type and the presence of tile drainage; (B) baseline or ‘existing’ wetland area (min-max = 0–940 km^2^) and potentially restorable wetland area (min-max = q0–478 km^2^); (C) nitrogen (N) fertilizer (total synthetic *+* organic) applications (min-max = 0–91 kg ha^−1^ yr^−1^) and initial soil organic N (total N; min-max = 23–44,000 kg ha^−1^ yr^−1^); (D) baseline predicted nitrate (${\text{NO}}_{3}^{-}$; min-max = 0–103 kg N ha^−1^ yr^−1^) and organic N yields (min-max = 0–90 kg N ha^−1^ yr^−1^). The color gradients are binned by quantiles. Supplementary material provides additional model input and calibration information.

We extended a recently developed wetland-integrated SWAT model for the URMB focused on river basin hydrology (Rajib *et al* 2020), updating the published version to simulate the response of ${\text{NO}}_{3}^{-}$ to river basin-scale wetland restoration scenarios. Novel to this study, we substantially modified model inputs (Text S2; figure 1) and agricultural management operations (Text S3), calibrated wetland N removal rates (see below and Text S6), and calibrated nitrate loads at 19 sites across the UMRB (Text S4; Tables S2–S5). The model was constructed to include 279 subbasins with a mean area of 1,600 km^2^ (SD = 1,100 km^2^), each having a single HRU depicting the subbasin’s dominant land use (figure 1(A)), soil, and slope.

Existing, or ‘baseline’, model wetlands (figure 1(B)) were identified as topographic depressions that intersected non-floodplain wetlands within the National Wetlands Inventory (Lane and D’Amico 2016, Wu and Lane 2016, US Fish & Wildlife Service 2018). From hereafter we refer to these non-floodplain wetlands as ‘wetlands’. Baseline wetlands covered ∼23,000 km^2^ of the basin (or ∼5% of UMRB area). Further information regarding physical wetland representations in the model is provided in Text S5 and Table S1. The complete, new model set-up resulted in baseline N inputs and outputs depicted in figures 1C and 1D.

Wetland ${\text{NO}}_{3}^{-}$ removal was simulated at a daily time-step per each subbasin (see Text S6). For each day, the subbasin’s wetlands received a constant fraction (specified as the fraction of land draining to the wetlands) of the subbasin’s simulated daily flow and nutrient load, comprising surface, shallow subsurface and groundwater contributions. Tile effluent could not be routed directly to wetlands in SWAT (similar to most existing large river basin models; see section 4.2). The wetland’s ${\text{NO}}_{3}^{-}$ removal (kg day^−1^) was then simulated as the product of the wetland’s daily simulated surface area (ha), the wetland’s daily simulated ${\text{NO}}_{3}^{-}$ concentration ($\left[{\text{NO}}_{3}^{-}\right]$; kg m−^3^), a user-specified and calibrated wetland N-removal constant (m yr^−1^), and a constant unit conversion factor (Neitsch *et al* 2011). The model’s N-removal constant is intended to account for denitrification and plant uptake of ${\text{NO}}_{3}^{-}$ of the aggregated wetland within each subbasin (Ikenberry *et al* 2017). Spillage and a corresponding $\left[{\text{NO}}_{3}^{-}\right]$ from a subbasin’s wetland to the nearest stream reach occurred whenever the wetland’s daily simulated storage exceeds the wetland’s maximum storage capacity.

### 2.2. Wetland restoration scenario analyses

We simulated change in ${\text{NO}}_{3}^{-}$ concentrations, yields, and loads at the outlets of the 279 subbasins and the UMRB in response to restoration of potentially restorable wetlands (Figure S3). Our restoration simulations considered only wetlands outside of floodplains (i.e., non-floodplain wetlands) because of their proximity to nonpoint pollution sources (Cheng *et al* 2020) and the capacity to classify their previous (historic) locations for restoration based on topographic analyses. Potentially restorable wetlands (figure 1(B)) were defined as topographic depressions (Wu and Lane 2016) that did not intersect current NWI non-floodplain wetlands, i.e., those not included in the baseline model. The depressions were assumed to indicate locations that may have historically functioned as wetlands but were drained or otherwise converted to support alternative land uses (primarily agricultural production within the UMRB). Upland areas that did not contain depressions were not considered as potential locations for wetland restoration. The potentially restorable wetlands totaled ∼8,000 km^2^ (or ∼2% of UMRB area) and ranged from 0 to 12% of subbasin areas.

We first developed and simulated a single model scenario depicting complete restoration of the basin’s potentially restorable wetlands. With this scenario, we assessed the maximum capacity of wetland restoration to reduce ${\text{NO}}_{3}^{-}$ levels at subbasin and river basin outlets. The scenario was constructed by revising the baseline model’s wetland representation to include the summed physical attributes (i.e., storage capacity, surface area, catchment area) of both the basin’s existing wetlands (used in the baseline calibrated model) and potentially restorable wetlands. This scenario increased the wetland maximum surface area from 0 to 478 km^2^ across the UMRB subbasins (mean = +28 km^2^). The additional maximum wetland storage capacity in the wetland restoration scenario ranged from 0 to 2.4 × 10^8^ m^3^ (mean = 1.6 · 10^7^ m^3^), and the proportion of subbasins draining to wetlands increased by 0 to 33% (mean = 4%). The model scenario also depicted a corresponding reduction in fertilizer application rates to account for the quantity of land that was assumed to be converted from agricultural production to restored wetland (Text S7).

We additionally ran a suite of 279 model scenarios, each targeting complete restoration of a single subbasin’s wetlands, in isolation. We hereafter term this approach subbasin ‘targeting’, i.e., targeting individual subbasins one-at-a-time for wetland restoration. For each scenario, we revised the baseline model’s description of wetlands in only the targeted subbasin to depict the summed physical attributes of the targeted subbasin’s existing and potentially restorable wetlands (Table S1). In turn, we reduced the quantity of fertilizer applied in only the targeted subbasin to account for the subbasin’s assumed conversion of land from agricultural production to restored wetland (Text S7).

The model scenarios – depicting either basin-wide or subbasin-targeted restoration – were compared against the baseline model, which incorporated representation of only existing or ‘baseline’ wetlands, to facilitate assessment of restoration effects in three ways. First, we simulated the daily effect of the restored wetlands on discharge (Q; mm yr^−1^), ${\text{NO}}_{3}^{-}$ yields (kg N ha^−1^ yr^−1^), and ${\text{NO}}_{3}^{-}$ concentrations (mg N l^−1^), which we averaged to and presented as mean annual changes at the UMRB outlet and the UMRB subbasin outlets. Second, we simulated the restored wetland ${\text{NO}}_{3}^{-}$ removal rates (kg N ha^−1^ yr^−1^) within each subbasin, which we calculated as the quotient of the mean annual ${\text{NO}}_{3}^{-}$ load reduction from each subbasin’s wetlands (kg N yr^−1^) and the area of restored wetlands (ha) in the subbasin. Lastly, we compared the magnitude of subbasin (or ‘local’) outlet ${\text{NO}}_{3}^{-}$ load reductions (kt N yr^−1^) from wetland restoration to ${\text{NO}}_{3}^{-}$ load reductions (kt N yr^−1^) at the UMRB outlet.

## 3. Results

### 3.1. River basin-scale nitrate reductions

**Figure 2.**
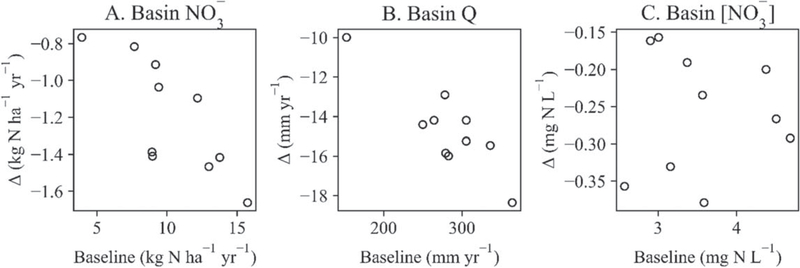
Yearly basin-wide wetland restoration impacts on simulated (A) nitrate yields, (B) discharge (Q), and (C) nitrate concentrations ($\left[{\text{NO}}_{3}^{-}\right]$) simulations at the UMRB outlet. The scatterplots showing simulated baseline (x-axes) and the difference (Δ) between wetland restoration values and baseline values (y-axes) at the basin outlet, for each year in the simulation period (2008–2017; n = 10).

Simulated basin-wide wetland restoration (8,000 km^2^ of potentially restorable wetlands, or 2% of the UMRB area) reduced the mean annual river basin outlet ${\text{NO}}_{3}^{-}$ load by 53 kt N yr^−1^, or by 12%. Approximately 93% of this load reduction was directly attributed to restored wetland storage and removal of ${\text{NO}}_{3}^{-}$; the remainder resulted from the accompanying reduction in fertilizer applications atop converted land (see Text S8). Basin-wide wetland restoration also reduced the mean annual ${\text{NO}}_{3}^{-}$ yield at the river basin outlet by 1.2 kg N ha^−1^ yr^−1^, with year-to-year yield reductions ranging from 0.77 to 1.66 kg N ha^−1^ yr^−1^ (figure 2(A)). Mean annual discharge (Q) at the basin outlet decreased by 15 mm yr^−1^ (5%), with year-to-year reductions ranging from 10 to 18.4 mm yr^−1^ (figure 2(B)). Year-to-year reductions in nitrate concentrations, hereafter symbolized as $\left[{\text{NO}}_{3}^{-}\right]$, at the river basin outlet fluctuated between 0.16 and 0.38 mg N L^−1^ (figure 2(C)), and the basin’s mean annual $\left[{\text{NO}}_{3}^{-}\right]$ declined by 0.25 mg N L^−1^ (7%).

### 3.2. Subbasin-scale nitrate reductions

**Figure 3.**
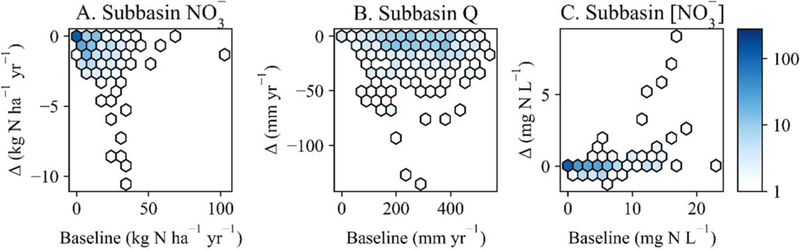
Average annual wetland restoration impacts on simulated subbasin-scale (A) nitrate yields, (B) discharge (Q), and (C) nitrate concentrations ($\left[{\text{NO}}_{3}^{-}\right]$). The point density plots show simulated baseline (x-axes) and the difference (Δ) between wetland restoration values and baseline values (y-axes) across subbasins (n=279). The color gradient for subfigures (A-C) show the number of subbasins represented by each hexagon: darker colors represent high point densities; lighter colors represent low point densities.

The effects of subbasin-targeted wetland restoration were varied. Restoration reduced ${\text{NO}}_{3}^{-}$ yields at the 279 subbasin outlets by 0 to 10.6 kg N ha^−1^ yr^−1^ and Q by 0 to 135.6 mm yr^−1^ (figure 3(A)–(B)). While restoration reduced $\left[{\text{NO}}_{3}^{-}\right]$ at a majority of subbasin outlets, it tended to increase $\left[{\text{NO}}_{3}^{-}\right]$ in subbasins with higher baseline $\left[{\text{NO}}_{3}^{-}\right]$ simulations (figure 3(C)), i.e., of the 25 subbasins with mean annual $\left[{\text{NO}}_{3}^{-}\right]⩾10\text{mg}{\text{NL}}^{-1}$, 23 (92%) had increased $\left[{\text{NO}}_{3}^{-}\right]$ following restoration. Changes in $\left[{\text{NO}}_{3}^{-}\right]$ from wetland restoration ranged from −1.25 to 9.1 mg N L^−1^ across subbasin outlets.

**Figure 4.**
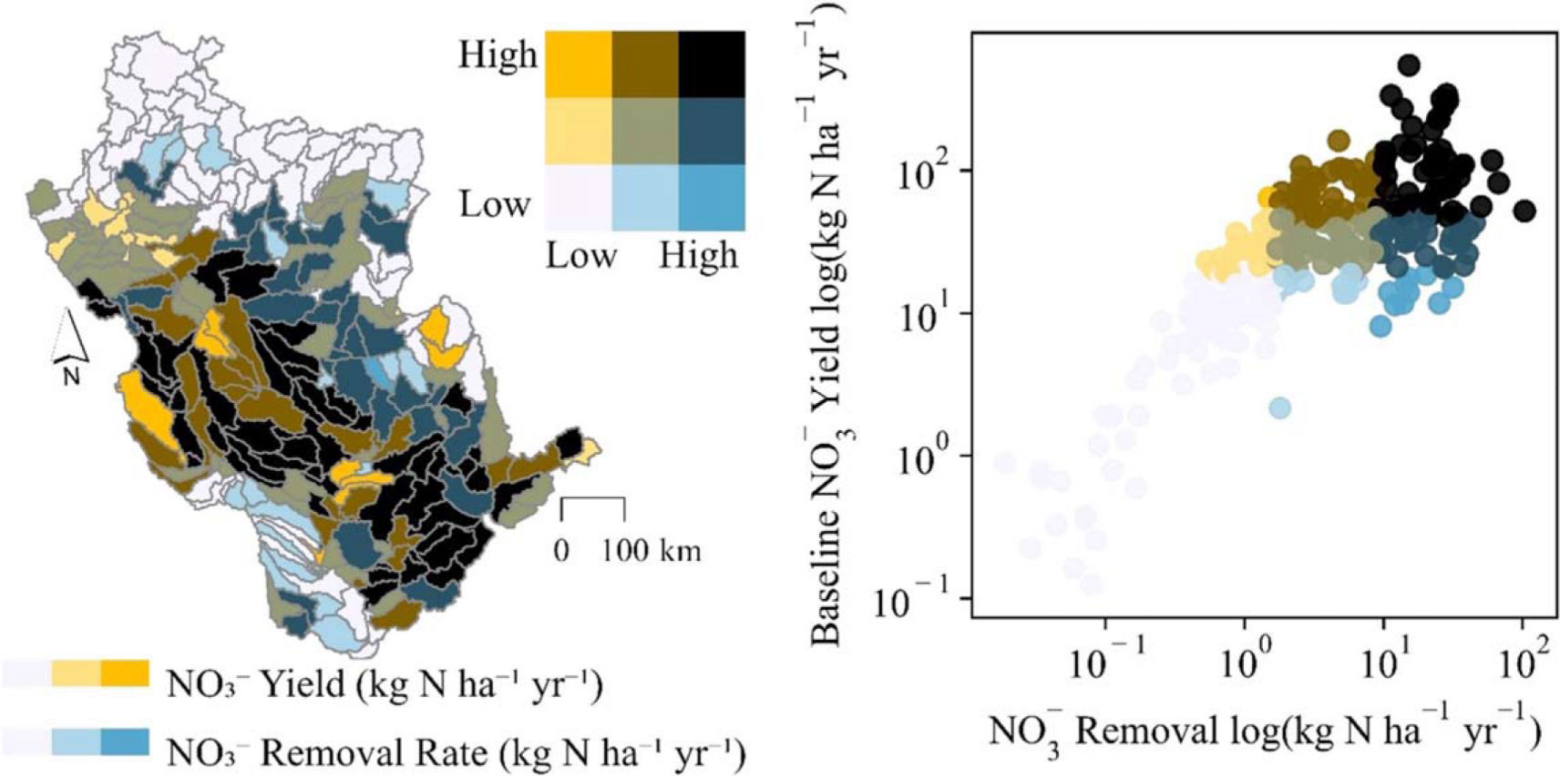
Restored wetlands generally removed ${\text{NO}}_{3}^{-}$ at higher average annual rates (min-max = 0–547 kg N ha^−1^ yr^−1^) in subbasins with higher average annual baseline ${\text{NO}}_{3}^{-}$ yields (min-max = 0–103 kg N ha^−1^ yr^−1^). The color scheme, binned by quantiles, is shared between the scatterplot and map.

Subbasin-targeted restoration revealed spatial variations in wetland ${\text{NO}}_{3}^{-}$ reductive capacity. Simulated wetland ${\text{NO}}_{3}^{-}$ removal rates ranged from 0.13 to 546 kg N ha^−1^ yr^−1^ across UMRB subbasins (mean = 50 kg N ha^−1^ yr^−1^). The restored wetlands removed ${\text{NO}}_{3}^{-}$ at higher rates in subbasins with higher baseline simulated ${\text{NO}}_{3}^{-}$ yields, i.e. the basin’s critical source areas or ‘hotspots’ (Evenson *et al* 2021); figure 4. These subbasins tended to be in the basin’s mid-section where agricultural activities and subsurface tile drainage are prevalent (figure 1(A), (D)). In fact, all subbasins above the 75th percentile ${\text{NO}}_{3}^{-}$ yields were simulated as tile drained agriculture.

**Figure 5.**
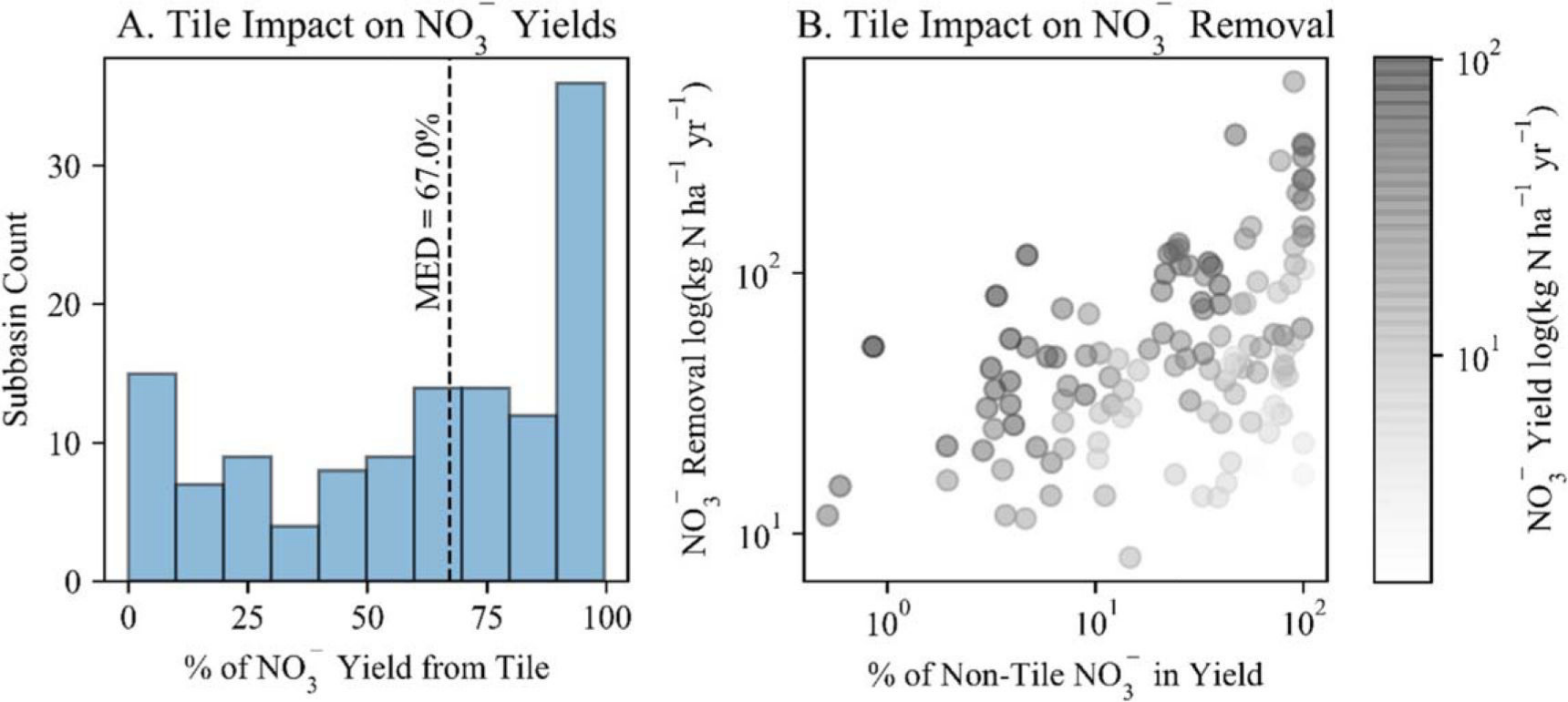
Tile drains were an important ${\text{NO}}_{3}^{-}$ loss pathway in tile drained subbasins (median percentage of total ${\text{NO}}_{3}^{-}$ yield contributed via tiles was 67% in these subbasins (A), and these drains effectively reduced wetland capacity to remove ${\text{NO}}_{3}^{-}$ because tile effluent did not enter the wetlands (B). In (B), ${\text{NO}}_{3}^{-}$ was removed at higher rates (moving further from the origin on the y-axis) as the percentage of non-tile ${\text{NO}}_{3}^{-}$ (i.e., ${\text{NO}}_{3}^{-}$ contributions from surface, shallow subsurface and groundwater flows) in the total ${\text{NO}}_{3}^{-}$ yield became larger (moving further from the origin on the x-axis), though this relationship was weaker for subbasins with lower magnitude total ${\text{NO}}_{3}^{-}$ yields (see lighter colored points).

Subsurface tile drains limited the restored wetlands’ ${\text{NO}}_{3}^{-}$ reductive capacity. Tile drains were an important pathway for ${\text{NO}}_{3}^{-}$ loss – or export – from tile drained subbasins; model structure prevented routing this effluent directly to wetlands. The median percentage of total subbasin ${\text{NO}}_{3}^{-}$ export via tiles was 67% (figure 5(A)). We accordingly observed that restored wetland ${\text{NO}}_{3}^{-}$ removal rates increased as the percentage of non-tile ${\text{NO}}_{3}^{-}$ (i.e., ${\text{NO}}_{3}^{-}$ contributions from surface, shallow subsurface and groundwater flows) in the total ${\text{NO}}_{3}^{-}$ yield became larger (figure 5(B)).

### 3.3. Relating subbasin-scale nitrate reductions to the river basin outlet

**Figure 6.**
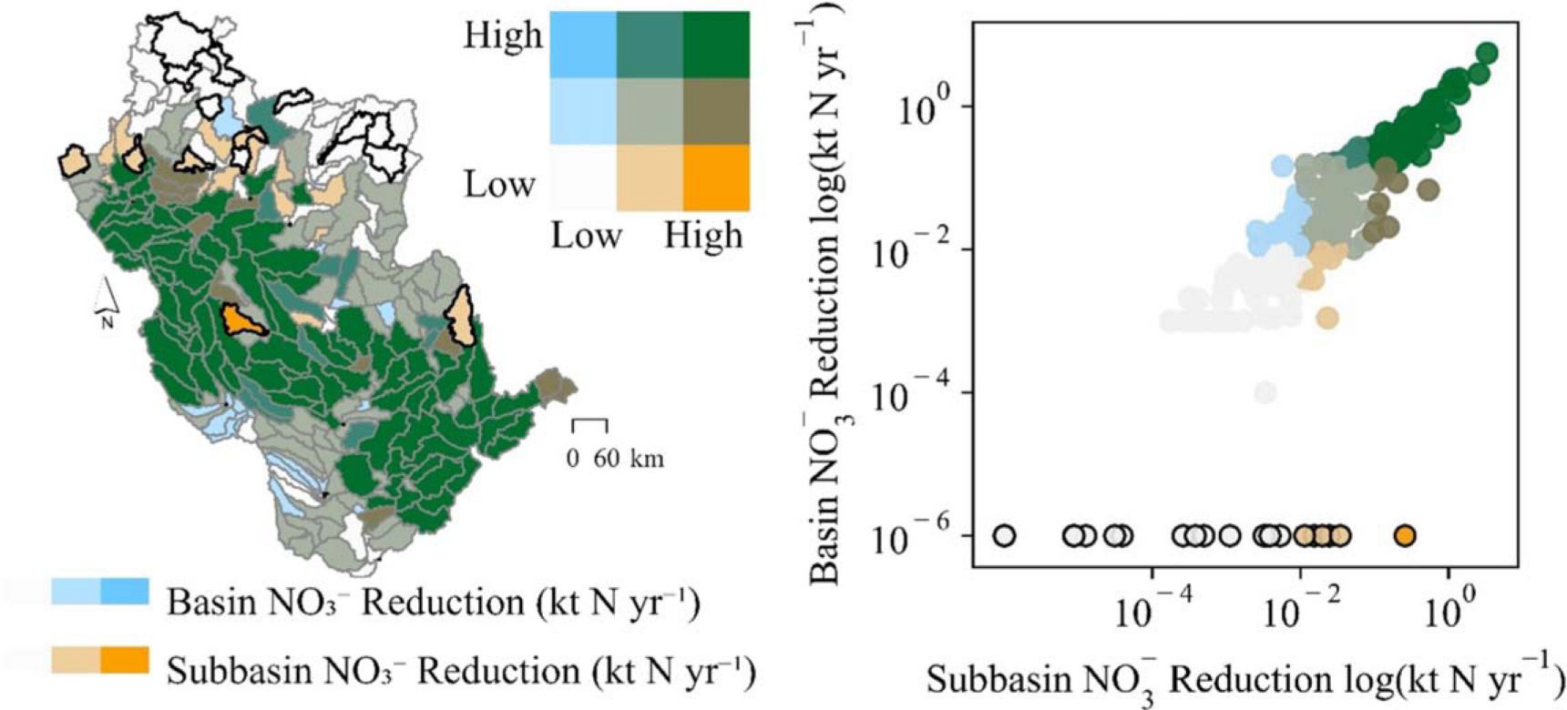
Subbasin scale ${\text{NO}}_{3}^{-}$ load reductions (min-max = 0–3.3 kt yr^−1^) did not always result in ${\text{NO}}_{3}^{-}$ load reductions of equivalent magnitude at the basin outlet (min-max = −0.069–5.5 kt yr^−1^), particularly at lower ${\text{NO}}_{3}^{-}$ reduction magnitudes. While linearly correlated (see scatterplot), the relationship between subbasin and basin outlet ${\text{NO}}_{3}^{-}$ load reductions only became strong at higher magnitudes of ${\text{NO}}_{3}^{-}$ load reductions. Subbasins in which simulated restoration reduced subbasin scale ${\text{NO}}_{3}^{-}$ loads but did not change or increased ${\text{NO}}_{3}^{-}$ loads at the basin outlet are outlined in black in both the scatterplot and map. The color scheme, binned by quantiles, is shared between the scatterplot and map.

We observed a clear linear relationship between wetland restoration-affected ${\text{NO}}_{3}^{-}$ load reductions at the subbasin outlets and those of the river basin outlet (figure 6). The relationship was strong at higher magnitude load reductions at the subbasin scale (moving towards the upper-right corner of the figure 6 scatterplot). However, approximately equivalent magnitude ${\text{NO}}_{3}^{-}$ load reductions at the basin outlet were less likely to occur if wetland restoration resulted in low-magnitude subbasin ${\text{NO}}_{3}^{-}$ load reductions. Restoration-affected subbasin scale load reductions resulted in no change or even minor increases in ${\text{NO}}_{3}^{-}$ loads at the basin outlet in 19, or 7%, of the subbasins (see figure 6 points and subbasins outlined in black). These subbasins were mostly in the northern portion of the basin, further from the basin outlet – affording long travel times through the river network and thereby elevated potential for storage and transformation processes to occur (Text S1) – and with a relatively high number of existing wetlands, as represented in the baseline model (figure 1).

## 4. Discussion

### 4.1. Implications for the science and management of wetland restoration

It is clear that wetlands can effectively remove nitrogen across landscapes (Johnston 1991, Cheng and Basu 2017). However, apart from recent empirical approaches that assume stationary watershed conditions (Hansen *et al* 2018, Cheng *et al* 2020), the cumulative effects of wetlands on downgradient aquatic nutrient conditions remain largely elusive (Golden *et al* 2019). Watersheds are nonstationary systems, requiring dynamic, process-based simulations to quantify the link between wetland restoration and watershed nutrient conditions, particularly across large river basins. Using this process-based approach, we found that wetland restoration across substantially reduces ${\text{NO}}_{3}^{-}$ yields and loads (and to a lesser degree, concentrations) across the UMRB – with caveats.

Restoration of 8,000 km^2^ of the URMB’s historic wetlands would lead to a 12% reduction in mean annual ${\text{NO}}_{3}^{-}$ loads at the river basin outlet. These benefits were achieved by an average 4% increase in subbasin area draining to wetlands (i.e., the fraction of land draining to wetlands following restoration) and without additional conservation practices (e.g., tile-effluent management, described more below). Our results suggest that higher magnitude load reductions may be attained via minor increases in the quantity of land draining to wetlands. We therefore underscore the capacity of wetlands to benefit water quality at river-basin scales (Golden *et al* 2019, Cheng *et al* 2020), in the greater Mississippi River Basin specifically (Mitsch *et al* 2001), in portions of the UMRB (Hansen *et al* 2018), and at smaller watershed-scales (Arheimer and Wittgren 2002, Czuba *et al* 2018).

Yet our results indicate that wetland restoration does not uniformly reduce ${\text{NO}}_{3}^{-}$ in subbasins across this large river basin. Additional or complementary management practices will be needed to meet N load reduction targets set for the greater Mississippi River Basin – and likely other large river basins. Our simulated 53 kt yr^−1^ (12%) reduction in mean annual ${\text{NO}}_{3}^{-}$ loads following restoration of 8,000 km^2^ of wetlands falls short of the 20% total nitrogen (TN) load reduction target set for the year 2025 (Hypoxia Task Force 2015) – noting that ${\text{NO}}_{3}^{-}$ comprised 86% of the baseline model mean annual TN load. These results also highlight a need for cost-benefit analyses (à la Singh *et al* (2019) and Hansen *et al* (2021)) to inform restoration decision making processes.

Further, our simulations provide a low estimate of the extent to which wetland restoration reduces river basin nitrogen loads at the outlet, compared to other recent studies. For example, Cheng *et al* (2020) predicted targeted wetland restoration resulting in a 467 kt yr^−1^ N load reduction for the greater Mississippi River Basin – meaning a 115 kt yr^−1^ decrease in N loads for the UMRB, assuming restoration of the same extent of potentially restorable wetland area considered in our analysis (see Text S9 and Table S2). This is almost double our simulated ${\text{NO}}_{3}^{-}$ load reductions in response to wetland restoration across the UMRB. Based on our findings, we suggest that wetland restoration should be implemented in concert with additional in-field conservation practices (e.g., cover crops), edge-of-field practices that treat tile-effluent (e.g., constructed wetlands or denitrifying bioreactors (Crumpton *et al* 2020)), or improved fertilizer management with reductions in fertilizer inputs to the basin.

Our methodological approach may, in part, explain our lower ${\text{NO}}_{3}^{-}$ reductions to wetland restoration relative to previously published studies (Mitsch *et al* 2001, Cheng *et al* 2020). Cheng *et al* (2020), for example, used the spatial distribution of wetlands and wetland nutrient removal rate estimates to calculate cumulative wetland removal potential – and this removal was assumed to be directly realized at the basin outlet as a steady-state basin condition. In contrast, our model simulated dynamic wetland ${\text{NO}}_{3}^{-}$ removal, estimated via the daily expansion and contraction of wetland surface area and daily changes in $\left[{\text{NO}}_{3}^{-}\right]$.

Our results also suggest potentially muted downstream impacts of wetland restoration because of landscape and in-stream N processing that is represented in the model (Text S1), highlighting the importance of considering these processes when assessing river basin-scale effects of wetland restoration. For example, tile drainage reduced wetland capacity to remove ${\text{NO}}_{3}^{-}$, figure 5; Text S3). Further, wetland restoration produced dissimilar subbasin and river basin outlet load reductions if subbasins had low-magnitude ${\text{NO}}_{3}^{-}$ decreases (figure 6) – and these differences resulted from the model’s simulation of in-stream N processes and removal of additional N (figure 6; Text S1).

While our findings demonstrate the benefits of targeted approaches to wetland restoration, we suggest a careful focus on processes that may limit wetland capacity to receive surplus nitrogen. We found restoration was more effective within the basin’s high N yield areas (figure 4), which lends credence to targeting approaches. However, restoration targeting these high N yield subbasins was less effective in subbasins with elevated ${\text{NO}}_{3}^{-}$ losses via tile drains (figure 5), enhanced by tile effluent not being routed to wetlands in the model. While wetlands are sometimes restored or constructed specifically to receive tile effluent (Crumpton *et al* 2020), our model reflects the UMRB as a whole: an expansively tiled landscape that promotes hydrologic connections and unmitigated nutrient transport. Thus, our results support targeted restoration of wetlands in high nitrogen yield or source areas but suggest that efforts may be less successful where tile-drains continue to bypass wetlands, limiting wetland capacity to receive and remove ${\text{NO}}_{3}^{-}$ from the landscape. Tile drainage should be considered in addition to other landscape characteristics and processes - e.g., climate change (Bosch *et al* 2014), and nutrient legacies (Van Meter *et al* 2018) - in prioritizing restoration efforts and projecting river-basin scale effects.

### 4.2. Opportunities for future research

River-basin scale simulations necessitate aggregating fine-scale processes that may affect restoration efficacy. For example, we simulated wetland NO_3_–N removal as a function of a N-removal constant, depicting the aggregate effect of wetland denitrification, settling, and plant-uptake processes per subbasin. Model simulations were highly sensitive to the N removal constant (p < 0.001; Table S3). While we calibrated the parameter to in-stream ${\text{NO}}_{3}^{-}$ loads, its specification could be alternatively verified using subbasin scale summaries of wetland N removal rates, when available – though we caution that individual wetland N removal rates may insufficiently represent aggregate wetland N removal per subbasin. Future research on aggregating or scaling-up individual wetland N removal rates will decrease uncertainty and improve confidence in model predictions.

Future analyses would benefit from a refined model representing interactive processes between tile-drainage and wetland restoration. A substantial portion of UMRB subbasins (49%) were simulated as tile-drained (figure 1(A)), and these drains were an important pathway for ${\text{NO}}_{3}^{-}$ losses (the mean percentage of total subbasin ${\text{NO}}_{3}^{-}$ losses via tiles was 60% [STD = 33%]). UMRB wetlands are often restored by ‘breaking’ existing tile lines to partially restore their hydrology. In addition, wetlands may be purposefully positioned downslope from fields to receive effluent from working tiles and more efficiently capture and filter nutrients (Zedler 2003, Crumpton *et al* 2020). SWAT (SWAT2012 ver. 659) does not currently facilitate representation of tile-effluent entering wetlands. Thus, our analysis will have underestimated the efficacy of restoration without tile-effluent routing to restored NFWs or at downslope treatment wetlands. We recommend structural modifications to the SWAT model to facilitate representation of these dynamics.

River-basin scale analyses of wetland restoration – and other water quality mitigation strategies, for that matter – relate a land management action (e.g., restoration) to nutrient loads and concentrations at an outlet-point separated by considerable distance. The storage and transformation of nutrient across these distances represents a gulf of uncertainty. Because a host of interacting nutrient storage and transformation processes are embedded in our model (see Neitsch *et al* (2011) and Text S1), our results are suggestive that nutrient dynamics along these pathways – i.e. between subbasins and the UMRB outlet – have important implications in determining the relative efficacy of restoration. We propose that there is a critical need to validate model simulation of these dynamics, particularly as applied at river-basin scales where they have greater capacity to impact simulation outputs. Subbasin scale summaries of river-lengths (e.g., initial nutrient storage, mineralization rates) could inform the parameterization and/or verification of the model’s simulation of in-stream nutrient dynamics.

## 5. Conclusions

We show that wetlands have the potential to substantially reduce ${\text{NO}}_{3}^{-}$ losses at large river basin scales, a novel finding at this spatial scale using a dynamic simulation approach. Our results suggest restoration efforts should be prioritized, or targeted, in subbasins with high nitrate yields. However, mediating processes (e.g., subsurface tile drains and in-stream storage and processing of N) may reduce wetland capacity to capture and remove ${\text{NO}}_{3}^{-}$ and affect change at the basin outlet – an important finding not yet revealed in previous studies. Specifically, our model simulated dynamic, intervening processes (e.g., subsurface tile drains and in-stream storage and processing of N) between the wetland and the subbasin, and river basin outlets. While it was outside the scope of this work to discern the exact in-stream processes controlling ${\text{NO}}_{3}^{-}$ dynamics, we highlight a need for additional process-based river basin scale analyses of wetland impacts downstream nutrient conditions.

We show that wetland restoration can be an effective means of reducing ${\text{NO}}_{3}^{-}$ loads to the lower Mississippi and will likely mitigate the Gulf of Mexico hypoxia. However, management would benefit from investments in other mitigation actions, based on our findings that wetland restoration alone in this highly modified and tiled large river basin would not meet load reduction targets. Wetland restoration may therefore serve as an effective pillar for overarching water quality mitigation plans that incorporate supplemental conservation actions (e.g., cover crops, tile-drainage mitigation, and strategic nutrient management).

## Supplementary Material

Supplementary data 7

Supplementary data 8

Supplementary data 6

Supplementary data 5

Supplementary data 3

Supplementary data 4

Supplementary data 2

Supplementary data 1

## Data Availability

The data that support the findings of this study will be openly available following an embargo via the Environmental Protection Agency Science Hub. Data will be available from 31 December 2021.
