# Supplementary material for: Wetland restoration yields dynamic nitrate responses across the Upper Mississippi river basin
Source: Environ Res Commun. Author manuscript; Available in PMC 2022 Jan 1. (PMC8567145; doi:10.1088/2515-7620/ac2125)
Supplement: Supplementary data 1 [file NIHMS1740368-supplement-Supplementary_data_1.pdf]

Supplemental Information

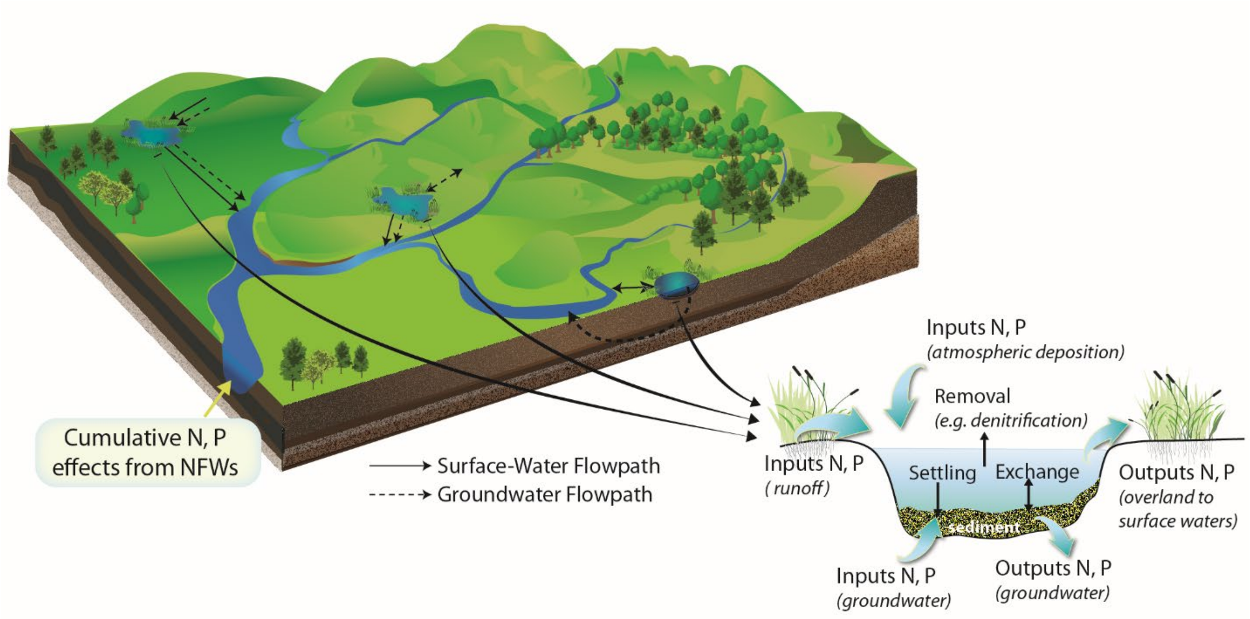

Figure S1. Conceptual model linking biogeochemical processes in individual wetlands (on right) to their cumulative effects on nutrients at the river basin outlet (left). Note that in this paper, we simulate N removal, settling, and benthic exchange in wetlands as one combined, or aggregate, removal rate constant. Reprinted with permission from Golden et al. Copyright (2019) American Chemical Society.

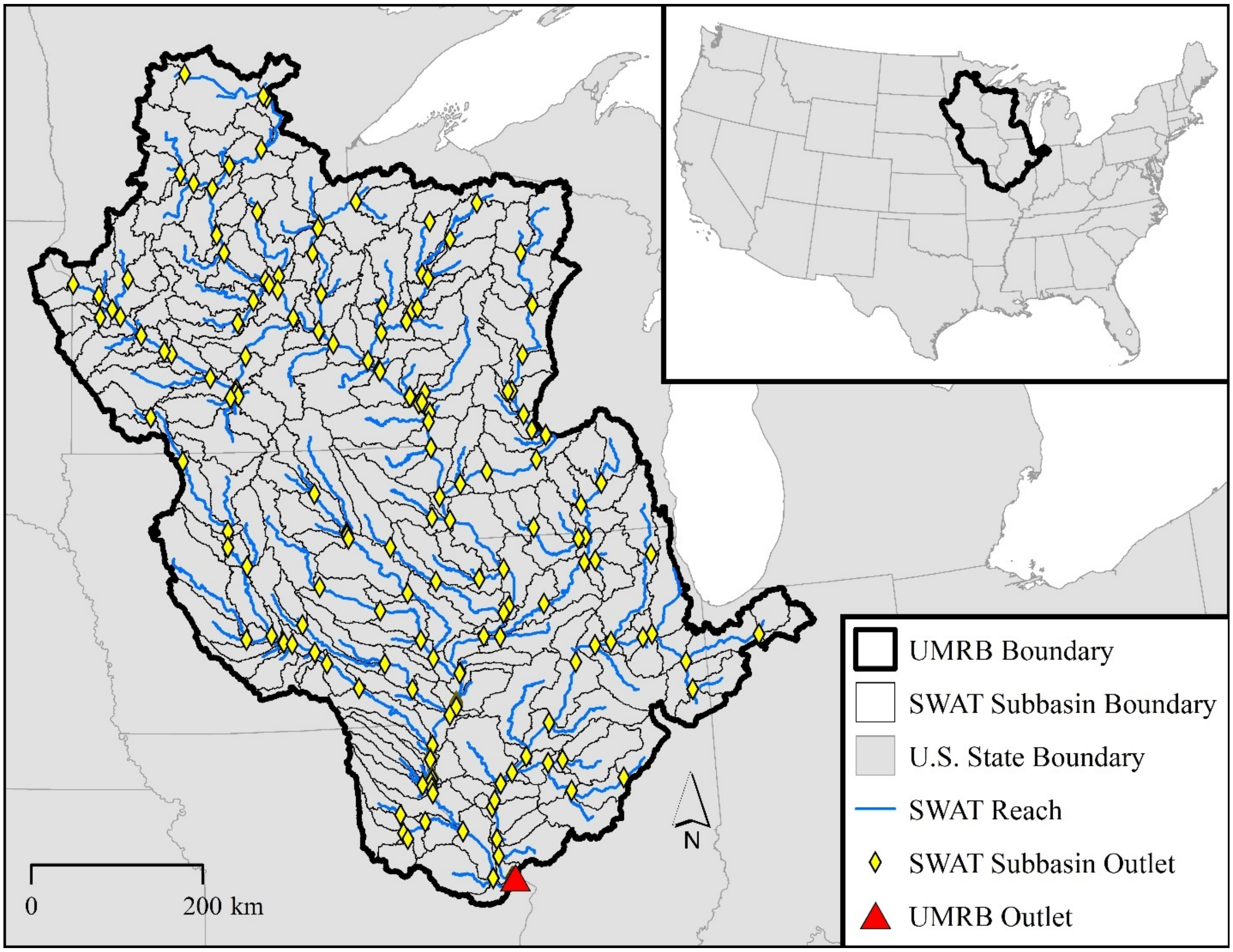

Figure S2. Upper Mississippi River Basin study/model simulation area, including model (SWAT) subbasins and associated stream reaches, subbasin outlets where changes in NO_3-N in response to wetland restoration were simulated and assessed, and the UMRB outlet, similarly where changes in NO_3-N in response to wetland restoration were simulated and assessed.

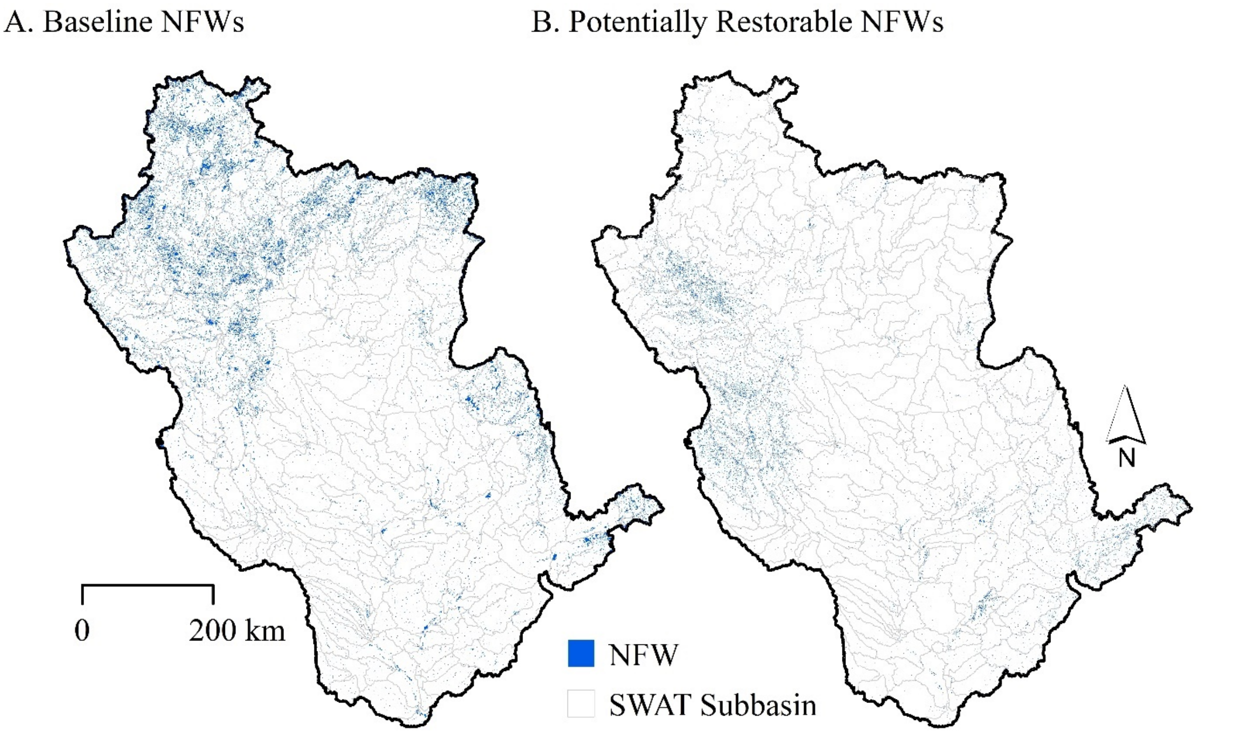

Figure S3. Spatial distribution (with model subbasins outlined in grey) of (A) the baseline, calibrated model non-floodplain wetlands (NFWs) and (B) potentially restorable NFWs used in the restoration analyses. The distribution of NFWs in the restoration analyses included (A) + (B).

Text S1: Overview of SWAT nutrient transformation and transport processes

The theoretical documentation for SWAT, including each governing equation for its land and in-stream simulations of hydrological and nutrient cycling processes can be found in Neitsch et al. (2011). In short, the land phase of the model simulates daily water balances and transport of water to the main subbasin. It also calculates the transformation and transport of several forms of nitrogen (and phosphorus). Transformation of nitrogen in the soil is controlled by partial ordinary differential equations that represent the nitrogen cycle (e.g., N_2 fixation, N plant uptake, denitrification, leaching, and volatilization). Nitrogen cycling in wetlands is simulated as described below (Text S4), which explains how wetland removal of NO_3-N (kg day^{-1}) is the product of daily product of the wetland's daily simulated surface area (ha), the wetland's daily simulated NO_3-N concentration ([NO_3^--N]) (kg m^{-3}), and a user-specified wetland N-removal constant (m yr^{-1}). Excess nitrate NO_3-N from wetlands or surface soils either infiltrates to deeper groundwater (which is subsequently transported to subbasin stream and ultimately to the watershed outlet) or is transported to the main subbasin reach through surface runoff or lateral subsurface flow.

In-stream water quality processes in SWAT are adapted from the QUAL2E model (Brown and Barnwell, 1987). SWAT calculates daily dissolved and sorbed nutrients in the stream and sediment, respectively, combined with transport equations that move the dissolved or particulate nutrients downstream to the basin outlet. In-stream transformation of nitrogen in the model is governed by partial differential equations representing growth and decay of algae, biological oxidation rates for different nitrogen species, water temperature, and settling of organic N wih sediment. Equations for algal growth as a limiting factor for nutrients and organic nutrient, ammonium, nitrite, and nitrate cycling within surface waters can be found in Section 7.3 of Neitsch et al. (2011).

We focused our analysis on NO_3^- because it is the dominant form of nitrogen (N) in managed agricultural landscapes (Crumpton et al. 2020). We assessed changes in NO_3^- concentrations and loads so that results are readily translatable to water quality targets (e.g., maximum contaminant levels under the US Safe Drinking Water Act; targeted load reductions for the Mississippi River Basin) and further focus on NO_3^- yields to estimate incremental contributions of wetland restoration to basin scale NO_3^- reductions. The main governing equation for nitrate transformation with the stream is:

$$\Delta\, NO3_{str} = (\beta_{N,2} * NO2_{str} - (1 - fr_{NH4}) * \alpha^1 * \mu_1 * algae) * TT$$

where $NO3_{str}$ is the daily change in nitrate concentration (mg N/L), $\beta_{N,2}$ is the biological oxidation rate constant of nitrite to nitrate (day^{-1}), $NO2_{str}$ is the nitrite concentration at beginning of the day (mg N/L), fr_{NH4} is the fraction of algal nitrogen uptake from the ammonium pool, α^1 is the fraction of algal biomass as nitrogen (mg N/mg algal biomass), is the local algal growth rate (day^{-1}), and TT is the travel time of flow in each subbasin reach segment (day or hour). For this work, we simulated in-stream processes as their default values in the model.

Text S2: Baseline model N input rates

We represented multiple nitrogen inputs to the landscape, including synthetic and organic (i.e., manure) nitrogen fertilizer (Figure 1C) and atmospheric nitrogen deposition inputs were specified from county-level fertilizer sales and livestock data and the Community Multiscale Air Quality (CMAQ) modeling system, respectively (Sobota et al. 2013; Pickard et al. 2015); see below). Initial soil organic nitrogen (Figure 1C) – which affected NO_3^- values via the model's simulation of nitrogen mineralization (see Neitsch et al. 2011) – was specified as a function of soil organic carbon estimates from the State Soil Geographic (STATSGO) dataset (Soil Survey Staff 2014). Nitrogen inputs via fixation were simulated if fertilizer inputs did not meet the nitrogen demands of planted legumes (i.e. soybeans) in agricultural subbasins. We assumed a negligible impact for point-source inputs – an assumption supported by Crawford et al. (2019).

We estimated the rate of fertilizer application that was applied in each subbasin under baseline conditions using spatial data obtained from the US EPA's EnviroAtlas data-catalog (Pickard et al. 2015). We obtained gridded (30-m resolution) spatial data depicting synthetic and organic N fertilizer (kg ha^{-1} yr^{-1}) application rates across the basin (Pickard et al. 2015). We multiplied each grid cell (depicting either synthetic or organic N) by the cell area (30x30-m = 0.09 ha) to determine the quantity of fertilizer applied per grid cell per year (kg yr^{-1}). We then overlaid the model's subbasin boundaries on the grids and summed all grid cell values within each subbasin to determine the aggregate quantity of fertilizer applied per subbasin per year (kg yr^{-1}). We then divided the aggregate quantity of fertilizer applied (kg yr^{-1}) within each subbasin by the subbasin's respective area (ha) to determine the subbasin's fertilizer application rate (kg ha^{-1} yr^{-1}).

We estimated the average annual rate of dry and wet deposition of nitrate and ammonium using spatial data describing mean annual deposition (kg N ha^{-1} yr^{-1}) per HUC-12, as obtained from the US EPA's EnviroAtlas data-catalog (Pickard et al. 2015). Because the SWAT model requires wet deposition inputs as concentrations (mg N L^{-1}), we multiplied each HUC-12's wet deposition rates (provided in kg N ha^{-1} yr^{-1} for both nitrate and ammonium) by the HUC-12's area (ha) to estimate average annual deposition of nitrate and ammonium (kg N yr^{-1}). We then divided these average annual estimates by each HUC-12's average annual precipitation (mm) (also obtained via the US EPA's EnviroAtlas data-catalog [Pickard et al. 2015]), giving a mean annual nitrate and ammonium (mg L^{-1}) wet deposition rate per HUC-12. We then overlaid SWAT subbasin boundaries on the HUC-12 boundaries and calculated area-weighted mean values of average annual dry and wet nitrate and ammonium deposition for each SWAT subbasin, which were applied as model inputs. The mean annual wet deposition rates (mg N L^{-1}) were then multiplied by the precipitation data used to force the model (via DAYMET; Thorton et al. 2017) to simulate wet deposition (kg ha^{-1} yr^{-1}).

Text S3: Baseline model agricultural management operations

We assumed a generic corn-soybean rotation for the model's agricultural subbasins (i.e. subbasins for which agricultural land was the dominant land use) following Golden et al. (2019), which is generally representative of the UMRB as a whole. In the first year of the rotation, we simulated tillage (April 18), synthetic fertilizer application (April 20), planting (corn, April 25), harvest (October 15), and organic fertilizer application (November 1). In the second year of the rotation, we simulated synthetic fertilizer application (April 20), planting (soybeans, May 5),

harvest (October 15), and organic fertilizer application (November 1). Though synthetic fertilizers would not in-reality be applied immediately prior to the planting a legume such as soybeans (which is capable of N-fixation), we simulated fertilizer application at that time-point to better represent the subbasin's overall nutrient balance.

We simulated a substantial portion of the basin (128 subbasins or 49% of total basin area) as tile-drained (Figure 1A; also see Rajib et al. (2020) for additional details on tile drainage sources). Subbasins with tiles were primarily located in the basin's mid-section where agricultural activities and subsurface tile drainage are prevalent (Figure 1A, Figure 1D). All subbasins above the 75th percentile baseline NO_3^- yields were simulated as tile drained agriculture. However, model limitations prevented us from routing tile effluent to wetlands. This is an important area for future model modifications.

Text S4: SWAT calibration

We calibrated and verified the UMRB SWAT model to monthly discharge (Q) and nitrate (NO_3^-) + nitrite (NO_2^-) as N load estimates across a 10-year period (2008-2014 for calibration, 2015-2017 for verification, with 2005-2007 as a warm-up period). The model was forced using total daily precipitation and mean daily temperature estimates from Daymet (Thornton et al. 2014).

We obtained daily mean Q observations ($m^3 s^{-1}$) at 39 USGS gauges and daily mean NO_3^- + NO_2^- as N concentration ($mg L^{-1}$) observations at 19 USGS gauges throughout the UMRB (Tables S4 and S5). We used the US Geological Survey's Load Estimator (LOADEST) program (Runkel et al. 2004) to estimate daily NO_3^-+NO_2^- loads and improve the temporal completeness of the observational data for 17 of the 19 NO_3^- gauges used for model calibration and verification (Table S4 and S5). Therefore, LOADEST data was used as observed data to calibrate and verify the SWAT models. For each gauge, we executed all available LOADEST model options (i.e., models 1-9) and then selected the LOADEST model with the lowest Akaike information criterion (AIC) value that met model performance criteria as specified within the LOADEST software package (i.e., percent bias [Bp] < +/- 25% and Nash-Sutcliffe Efficiency [NSE] > 0). We did not apply LOADEST at gauge locations at which no LOADEST model met the performance criteria (subbasins 166 and 191, Table S6). The mean Bp and NSE values were 2.52% and 0.79, respectively, across the 17 gauge locations for which we applied LOADEST.

The daily Q and NO_3^-+NO_2^- load estimates were then aggregated to a monthly time-step and used as observed data for model calibration and verification. We assumed that NO_2^- constituted a marginal fraction of the NO_3^-+NO_2^- load observations and calibrated the model's NO_3^- simulations to the NO_3^-+NO_2^- load observations. We used a stepwise upgradient to downgradient calibration procedure (i.e., we calibrated upgradient gauges first and proceeded downgradient through the basin) to fit model parameters to the monthly Q and NO_3^- load observations. We identified 35 model parameters (Table S3) to be included in this procedure via literature review (White and Chaubey 2005; Golden et al. 2019).

We used model-suggested ranges for all parameters with exception for the aggregate wetland N-removal constant, for which a range of 0 to 40 $m yr^{-1}$ was used following (Ikenberry et al. 2017). We began the calibration procedure by fitting the model's basin scale parameters (i.e. parameters for which one value must be specified for the whole of the basin) to the Q and NO_3^- load observations. Next, and moving sequentially from upgradient-to-downgradient NO_3^- gauges, we fit the remaining model parameters to observations and estimates at each NO_3^- gauge

as well as all Q observations within the NO_3^- gauge's immediate upgradient catchment (see Table S7). The final calibrated UMRB SWAT model had mean Kling-Gupta Efficiency (KGE; Gupta et al. 2009) scores – weighted per each gauge's total upgradient drainage area – of 0.72 and 0.64 for Q during the calibration and verification periods, respectively; and 0.55 and 0.51 for NO_3^- loads during the calibration and verification periods, respectively (see Tables S4 and S5).

Text S5: Wetland physical attribute parameterization

We simulated individual wetland hydrology and nitrate removal for one aggregated wetland per subbasin. This means that each subbasin had one wetland with a surface area, maximum storage capacity, and catchment area (i.e., the fraction of subbasin land area that drains to the wetland) representing the sums for all of its constituent wetlands. Each subbasin's wetland maximum surface area extent was estimated as the total area of baseline wetlands within the subbasin (range = 0 to 938 km^2 across subbasins, mean = 77 km^2). Each subbasin's wetland maximum storage capacity was estimated using the topographic defined volume of the subbasin's baseline wetlands (via Wu et al. 2016); range = 0 to 1.06×10^9 m^3, mean = 8.18×10^7 m^3), and each subbasin's wetland catchment area (range = 0 to 1, mean = 0.804; values represent fractions of subbasin area) was estimated via topographic analysis of a 30-m resolution digital elevation model in ArcGIS (v10.5).

Specifically, SWAT facilitates description of the physical attributes each subbasin's wetlands via the WET_NSA, WET_MXSA, WET_NVOL, WET_MXVOL parameters, which describe the normal ('N') and maximum ('MX') surface area ('SA') (ha), and the normal and maximum volume ('VOL') ($m^3 \times 10^4$) of the wetland, respectively. We specified values for the maximum surface area (WET_MXSA) and volume (WET_MXVOL) of the wetland via analysis of wetland and depressional spatial data, while we assumed that the normal surface area (WET_NSA) and volume (WET_NVOL) was half (i.e. 50%) of their respective maximums.

For the baseline model, each subbasin's WET_MXSA and WET_MXVOL parameters were specified as the surface area and calculated volumes (via Wu and Lane 2016) of all surface depressions (via Wu and Lane 2016) that intersected non-floodplain wetlands in the National Wetland Inventory (NWI; via Lane and D'Amico, 2016) within the subbasin. For the restoration scenario, each subbasin's WET_MXSA and WET_MXVOL parameters were specified as the surface area and calculated volumes (via Wu and Lane 2016) of all surface depressions (via Wu and Lane 2016) within the subbasin.

The WET_FR parameter (i.e., wetland catchment area) in SWAT is the portion of each subbasin that drains the subbasin's wetlands via. We specified the WET_FR parameter in the baseline model as the portion of subbasin area that drains to the baseline wetlands; and specified the WET_FR parameter under restoration conditions as the portion of subbasin area that drains to the restored wetlands. These portions were calculated via topographic analysis of a 30-m resolution digital elevation model (DEM) in ArcGIS (v10.5). Table S3 lists the values assigned to the parameters described in this section.

Text S6: Wetland nitrogen removal constants

The Soil and Water Assessment Tool (SWAT) model simulates wetland removal of NO_3-N (kg day^{-1}) as the product of the wetland's daily simulated surface area (ha), the wetland's daily simulated NO_3-N concentration ([NO_3^--N]) (kg m^{-3}), and a user-specified wetland N-removal

constant (m yr^{-1}) (Neitsch et al. 2011). The wetland's N-removal constant is specified in the model's '*pnd*' inputs files as the NSETLW1 and NSETLW2 parameters. The model uses either the NSETLW1 or NSETLW2 parameters in the aforementioned NO_3-N removal calculation during different months of the year to represent conditions when biogeochemical interactions would be higher: for months IPND1 (March) through IPND2 (October) the model uses NSETLW1 and for months other than IPND1 through IPND2 the model uses NSETLW2. We calibrated both the NSETLW1 and NSETLW2 parameters (see Table S3).

The daily budget for NO_3-N in the wetland is then calculated by (Neitsch et al. 2011):

$$V * dc/dt = W(t) - Q * c - v * c * A_s$$

Where V is the wetland volume (m^3 H_2O), c is the NO_3-N concentration (kg/m^3 H_2O), dt is the time step (1 day), W_t is the load of NO_3-N entering the wetland (kg/day), Q is the water outflow rate from the wetland (m^3 H_2O /day), v is the N removal constant (as a velocity; m/day), and A_s is the area of the sediment/water interface (m^2).

Text S7: Scenario fertilizer application rate reductions

We estimated the rate of fertilizer application that was applied in each subbasin under restoration (i.e. scenario) conditions as:

$$F_{r,s} = \frac{F_{b,s} * (A_s - A_w)}{A_s}$$

where $F_{r,s}$ is the fertilizer application rate (kg ha^{-1} yr^{-1}) for subbasin s under restoration (r) (i.e. scenario) conditions, $F_{b,s}$ is the fertilizer application rate (kg ha^{-1} yr^{-1}) for subbasin s under baseline conditions, A_s is the area (ha) of subbasin s, and A_w is the area of restored wetland added to the subbasin s.

Text S8: Wetland vs. fertilizer application reduction impacts on nitrate simulations

We assessed change in NO_3^- concentrations, yields, and loads at subbasin and basin scales as the combined effect of an increased quantity of wetlands and the associated reduced fertilizer application (to account for the conversion of cropland to restored wetlands and the associated reduction fertilizer application). We executed two supplemental model scenarios to isolate the effects of the wetlands versus the fertilizer reduction. In the first scenario, we simulated complete restoration of the basin's non-floodplain wetlands (NFWs) but did not reduce the quantity of applied fertilizer as we did for the restoration scenarios presented in the main text. Hence, simulated NO_3^- reductions from this scenario (evaluated against the baseline model, as presented in the main text) were fully attributable to the wetlands.

In the second scenario, we reduced fertilizer application at the same magnitude as we did for the restoration scenario presented in the main text but did not simulate an increased quantity of wetlands. Therefore, simulated NO_3^- reductions from this scenario (evaluated against the baseline model, as presented in the main text) were fully attributable to the fertilizer reduction.

These scenarios indicated that the mean annual NO_3^- load at the basin outlet declined by 48.9 kt yr^{-1} due to the wetlands and 4.4 kt yr^{-1} due to the fertilizer reduction. The restoration scenario presented in the main text (assessing the aggregate impact of the wetlands and the fertilizer reduction) indicated that the mean annual NO_3^- load at the basin outlet would decline by 52.7 kt yr^{-1}. Therefore, ~93% of the simulated NO_3^- load reduction was attributable to the wetlands while ~8% was attributable to the fertilizer reduction.

Text S9: Cheng et al. (2020) comparison

Cheng et al. (2020) executed their analysis for the contiguous United States (while providing results for the Mississippi River Basin [MRB], specifically) though our study was limited to the Upper Mississippi River Basin (UMRB). It was therefore necessary to downscale the results presented by Cheng et al. (2020) to facilitate inter-study comparison. Cheng et al. (2020) determined a wetland area normalized wetland nitrogen (N) removal rate of 146 kg ha^{-1} yr^{-1} for restored wetlands in the MRB. The 146 kg ha^{-1} yr^{-1} removal rate was calculated as the quotient of the quantity of additional N removed by the restored wetlands (467 kt yr^{-1}; see Cheng et al. [2020] Extended Data Table 1) and the 31,971 km^2 of restored wetlands in the MRB (i.e., a 22% increase [see Cheng et al. [2020], pg. 629] over the MRB's 145,324 km^2 of existing wetlands [see Cheng et al. [2020] Extended Data Table 1]). Cheng et al. (2020) would therefore predict a 115 kt yr^{-1} (22%) reduction in the mean annual TN load at the UMRB outlet (525 kt yr^{-1} – [[7,896 km^2 x 100 x 146 kg ha^{-1} yr^{-1}] / 1,000,000] = 410 kt yr^{-1}), assuming (1) a baseline simulated mean annual total nitrogen (TN) load of 525 kt yr^{-1} at the UMRB outlet (as simulated by this study's baseline Soil and Water Assessment Tool [SWAT] model), and (2) 7,896 km^2 of restorable wetlands within the UMRB (as determined by this study's analysis of potentially restorable non-floodplain wetlands). See Table S2 for further information regarding our inter-study comparison.

References

Brown, LC and Barnwell, TO, Jr. 1987. The enhanced water quality models QUAL2E and QUAL2E-UNCAS documentation and user manual. EPA document EPA/600/3-87/007. USEPA, Athens, Georgia, USA.Cheng, F.Y., Van Meter, K.J., Byrnes, D.K. and Basu, N.B., 2020. Maximizing US nitrate removal through wetland protection and restoration. Nature, pp.1-6.

Crawford, J. T., et al. (2019). "Network controls on mean and variance of nitrate loads from the Mississippi River to the Gulf of Mexico." Journal of Environmental Quality 48(6): 1789-1799.

Crumpton, W. G., G. A. Stenback, S. W. Fisher, J. Z. Stenback and D. I. Green (2020). "Water quality performance of wetlands receiving nonpoint-source nitrogen loads: Nitrate and total nitrogen removal efficiency and controlling factors." Journal of Environmental Quality 49(3).

Golden, H.E., Rajib, A., Lane, C.R., Christensen, J.R., Wu, Q. and Mengistu, S., 2019. Non-floodplain wetlands affect watershed nutrient dynamics: A critical review. Environmental Science & Technology, 53(13), pp.7203-7214.

Gupta, H. V., et al. (2009). "Decomposition of the mean squared error and NSE performance criteria: Implications for improving hydrological modelling." Journal of hydrology 377(1-2): 80-91.

Ikenberry, C. D., et al. (2017). "Evaluation of existing and modified wetland equations in the SWAT model." JAWRA Journal of the American Water Resources Association 53(6): 1267-1280.

Lane, C.R. and D'Amico, E., 2016. Identification of putative geographically isolated wetlands of the conterminous United States. JAWRA Journal of the American Water Resources Association, 52(3), pp.705-722.

Neitsch, S. L., et al. (2011). Soil and water assessment tool theoretical documentation version 2009, Texas Water Resources Institute.

Pickard, B. R., Daniel, J., Mehaffey, M., Jackson, L. E., & Neale, A. 2015. EnviroAtlas: A new geospatial tool to foster ecosystem services science and resource management. Ecosystem Services, 14, 45-55

Rajib, A., et al. (2020). "Surface depression and wetland water storage improves major river basin hydrologic predictions." Water Resources Research 56(7): e2019WR026561.

Runkel, R. L., et al. (2004). Load Estimator (LOADEST): A FORTRAN program for estimating constituent loads in streams and rivers.

Sobota, D. J., et al. (2013). "Reactive nitrogen inputs to US lands and waterways: how certain are we about sources and fluxes?" Frontiers in Ecology and the Environment 11(2): 82-90.

Soil Survey Staff (2014). "Natural Resources Conservation Service, United States Department of Agriculture, US General Soil Map (STATSGO2)."

Thornton, P. E., et al. (2014). Daymet: Daily surface weather data on a 1-km grid for North America, Version 2, Oak Ridge National Lab.(ORNL), Oak Ridge, TN (United States).

White, K. L. and I. Chaubey (2005). "Sensitivity analysis, calibration, and validations for a multisite and multivariable SWAT model 1." JAWRA Journal of the American Water Resources Association 41(5): 1077-1089.

Wu, Q. and Lane, C.R., 2016. Delineation and quantification of wetland depressions in the Prairie Pothole Region of North Dakota. Wetlands, 36(2), pp.215-227.
